# REL-1017 (Esmethadone) Increases Circulating BDNF Levels in Healthy Subjects of a Phase 1 Clinical Study

**DOI:** 10.3389/fphar.2021.671859

**Published:** 2021-04-28

**Authors:** Sara De Martin, Daniela Gabbia, Franco Folli, Francesco Bifari, Paolo Fiorina, Nicola Ferri, Stephen Stahl, Charles E. Inturrisi, Marco Pappagallo, Sergio Traversa, Paolo L. Manfredi

**Affiliations:** ^1^ Department of Pharmaceutical and Pharmacological Sciences, University of Padova, Padova, Italy; ^2^ Department of Health Science, University of Milan, Milan, Italy; ^3^ Department of Medical Biotechnology and Translational Medicine, University of Milan, Milan, Italy; ^4^ Nephrology Division, Boston Children’s Hospital and Harvard Medical School, Boston, MA, United States; ^5^ Department of Psychiatry, University of California, San Diego School of Medicine, La Jolla, CA, United States; ^6^ Neuroscience Education Institute, San Diego, CA, United States; ^7^ Relmada Therapeutics, Inc., New York, NY, United States

**Keywords:** esmethadone, BDNF, depression, clinical trial, NMDAR

## Abstract

Brain-derived neurotrophic factor (BDNF), a neurotrophin widely expressed in the central nervous system, exhibits important effects on neural plasticity. BDNF has been implicated in the mechanism of action of ketamine, a N-methyl-d-aspartic acid receptor (NMDAR) antagonist with rapid anti-depressant effects in humans. REL-1017 (esmethadone), the d-optical isomer of the racemic mixture d-l-methadone, is devoid of clinically relevant opioid activity at doses expected to exert therapeutic NMDAR antagonistic activity in humans. The present study was conducted to ascertain the effects of oral administration of 25 mg of REL-1017 for 10 days on plasma BDNF in healthy subjects confined to an inpatient unit for a phase 1 clinical trial. We observed an increase in post-treatment BDNF plasma levels compared to pre-treatment levels. Post-treatment, Day 10 BDNF plasma levels ranged from 2 to 17 times pre-treatment levels in the 25 mg REL-1017 treatment group, whereas in the placebo group, BDNF plasma levels remained unchanged (*p* = 0.028). Diastolic blood pressure decreased significantly in subjects treated with REL-1017, while no effect could be observed in the placebo group. In conclusion, the administration of 25 mg REL-1017 significantly increased BDNF plasma levels and significantly decreased diastolic blood pressure in healthy subjects confined to an inpatient unit for a phase 1 clinical trial.

## Highlights:

- REL-1017 induces a significant and rapid increase in plasma BDNF levels in healthy subjects confined to an inpatient unit for a phase 1 clinical study.
- REL-1017 reduced diastolic blood pressure in these subjects.

## Introduction

Brain-derived neurotrophic factor (BDNF) is a neurotrophin that plays a key role in neural plasticity. In the central nervous system (CNS), BDNF is produced by neurons in different brain areas (Miranda et al., 2019) and binds to tyrosine kinase coupled receptors eliciting downstream effects. BDNF production has been implicated in the mechanism of action of antidepressant drugs (Li et al., 2010). In particular, the therapeutic effects of ketamine (Swainson et al., 2019), an established N-methyl-D-aspartic acid receptor (NMDAR) channel blocker with rapid antidepressant effects, are BDNF dependent (Pochwat et al., 2014; Kraus et al., 2019). In patients with treatment-resistant depression (TRD), ketamine rapidly and significantly increased plasma BDNF levels in treatment responders compared to non-responders (Haile et al., 2014). Similar results have also been obtained in preclinical studies, since ketamine and other NMDAR channel blockers produce rapid behavioral antidepressant-like effects in animal models. These effects are dependent on rapid synthesis of BDNF (Li et al., 2010; Autry et al., 2011). Furthermore, it has been observed that subjects carrying the Val66Met (rs6265) single nucleotide polymorphism (SNP), which has been shown to affect intracellular trafficking and secretion of BDNF, are at higher risk for developing major depressive disorder (MDD) compared to Val/Val subjects (Laje et al., 2012). This clinical observation is confirmed by animal studies, since the mouse model carrying this SNP in both alleles (BDNF^Met/Met^) replicates the phenotypic hallmarks of MDD. Furthermore, these authors demonstrated that BDNF was expressed in mouse brains at normal levels, but its neuronal secretion was defective. From the behavioral point of view, BDNF^Met/Met^ mice subjected to stressful conditions exhibited increased anxiety-related behaviors which were unresponsive to antidepressants (see (Tsai, 2018) and refs therein). Accordingly, Val/Val subjects were more likely to exhibit increased an antidepressant response to ketamine than Met carriers (Laje et al., 2012). Based on these observations, Liu and collaborators (Liu et al., 2012) hypothesized that the reduced antidepressant response to ketamine infusion observed in approximately 30% of treated patients might be related to the Val66Met SNP.

REL-1017 (esmethadone; dextromethadone), the dextro isomer of racemic d-l-methadone, is a low affinity NMDAR channel blocker with a half maximal inhibitor concentration (IC_50_) in the micromolar range, similarly to ketamine and dextromethorphan (Laurel Gorman et al., 1997; Hanania et al., 2020). In preclinical studies, REL-1017 improved the depressive behavior in rodent models of depression (Fogaça et al., 2019; Hanania et al., 2020) and increased levels of synaptic proteins in the medial prefrontal cortex (Fogaça et al., 2019). Mechanistic studies indicated that the antidepressant-like effects of REL-1017 observed in rodent models were mediated by mammalian target of rapamycin complex 1 (mTORC1) and BDNF induction of neural plasticity (Fogaça et al., 2019). In phase 1 clinical trials, REL-1017 demonstrated favorable safety, tolerability, and pharmacokinetic profiles (De Martin et al., 2018; Bernstein et al., 2019). Therefore, based on preclinical *in vivo* results (Fogaça et al., 2019) and on *in vitro* experimental data obtained with ketamine and other NMDAR channel blockers (Kotermanski and Johnson, 2009), we hypothesized that REL-1017 may preferentially block tonically and pathologically hyperactive NMDARs while sparing physiological phasic glutamatergic activity, allowing for improvement of depressive symptoms in the absence of cognitive side effects typical of NMDAR channel blockers. In this context, the down-regulation of excessive Ca^2+^ influx *via* tonically and pathologically hyperactive NMDARs would determine downstream effects of enhanced production/release of BDNF and restore BDNF-dependent neural plasticity.

Healthy volunteers undergoing voluntary hospitalization to test new pharmaceutical compounds with potential psychiatric indications likely are subjected to clinically meaningful stress. As with animal models of depression, excessive stress may cause molecular changes that underlie the development of depression, and these molecular changes may be reversed by REL-1017, *via* BDNF dependent mechanism, potentially determined by blocking excessive Ca^2+^ influx *via* hyperactive NMDARs (Fogaça et al., 2019). However, whether the therapeutic effects of REL-1017 in humans are correlated with enhanced production/release of BDNF remains to be determined. Furthermore, low circulating BDNF levels have been associated with coronary artery disease (CAD) (Lee et al., 2017), and a prospective study suggests that low BDNF concentrations predict CAD and higher all-cause mortality (Jiang et al., 2011). Therefore, low BDNF levels may be central to the crosstalk between CNS disorders and cardiovascular homeostasis. Furthermore, effects of stress on blood pressure (BP) have been demonstrated, and it is likely that exposure to stress may be linked to sustained BP elevations (Chrousos, 2009).

In light of these considerations, we evaluated the effects of REL-1017 administration on BDNF plasma levels, and systolic and diastolic BP in healthy volunteers confined to an inpatient unit for a phase 1 clinical study.

## Methods

### Subjects and Study Design

The present study was conducted as part of a single site, randomized, double-blind, placebo-controlled phase 1 clinical trial (ClinicalTrials.gov Identifier: NCT03637361) of 25 mg REL-1017 administered orally for 10 days to healthy volunteers admitted for 14 days to a Clinical Research Unit (CRU) (Bernstein et al., 2019). The 8 subjects, whose demographic characteristics are listed in Table 1, were randomly assigned to the 2 experimental groups, i.e., placebo (2 subjects) and 25 mg of REL-1017 (6 subjects). The demographic and physical characteristics were similar between the two treatment groups. BDNF plasma levels were measured before the first dose (Day 1, predose) and at Days 2, 6, and 10. Blood pressure was measured both 2 and 24 h after the administration of REL-1017.

**TABLE 1:** Demographic characteristics of the subjects enrolled in the study.

	Placebo (n = 2)	REL-1017 (n = 6)	*p* Value
Age (years)	38 ± 18	39 ± 8	Ns
Sex	2 females	3 males, 3 females	
BMI	26.7 ± 3.01	26.2 ± 2.68	Ns

Data are expressed as mean ± S.D.

### Measurement of BDNF Plasma Concentrations

Blood sampling and plasma REL-1017 concentrations, as well as BP measurements, were assessed as described by Bernstein et al. (2019). BDNF plasma levels were assessed in blood samples obtained before treatment start (Day 1, predose) and 4 h after administration of a 25 mg-dose of REL-1017 (six patients) or placebo (two patients) on Days 2, 6, and 10. Plasma levels of BDNF were measured with an ELISA kit (Raybiotech, Peachtree Corners, Georgia, United States) following the manufacturer’s instructions. Quantitative determination of BDNF was carried out by standard calibration curves obtained with human recombinant BDNF at concentrations ranging from 0.066 to 16 ng/ml (n = 7), processed following the same protocol used for the plasma samples. As expected, the calibration curves fitted an allosteric sigmoidal equation (*r* ^2^ ≥ 0.99). We first performed a preliminary study on the subjects’ plasma samples to find the appropriate dilution to obtain BDNF values within the calibration range (1:2 or 1:10 dilutions were selected as appropriate). Each concentration was the result of three independent determinations. Data are presented as mean ± SD or SEM.

### Statistical Analysis

Statistical analyses were performed with GraphPad Prism 8.0. Wilcoxon Signed Rank test was used to compare BDNF concentrations before treatment and 4 h after administration of REL-1017 or placebo at Days 2, 6, and 10. We also compared demographic characteristics and BDNF values obtained in placebo and treated subjects with Mann-Whitney test and assessed the existence of a correlation between plasma REL-1017 and BDNF concentrations by the Spearman correlation analysis. A *p*-value <0.05 was considered statistically significant.

## Results

**FIGURE 1:**
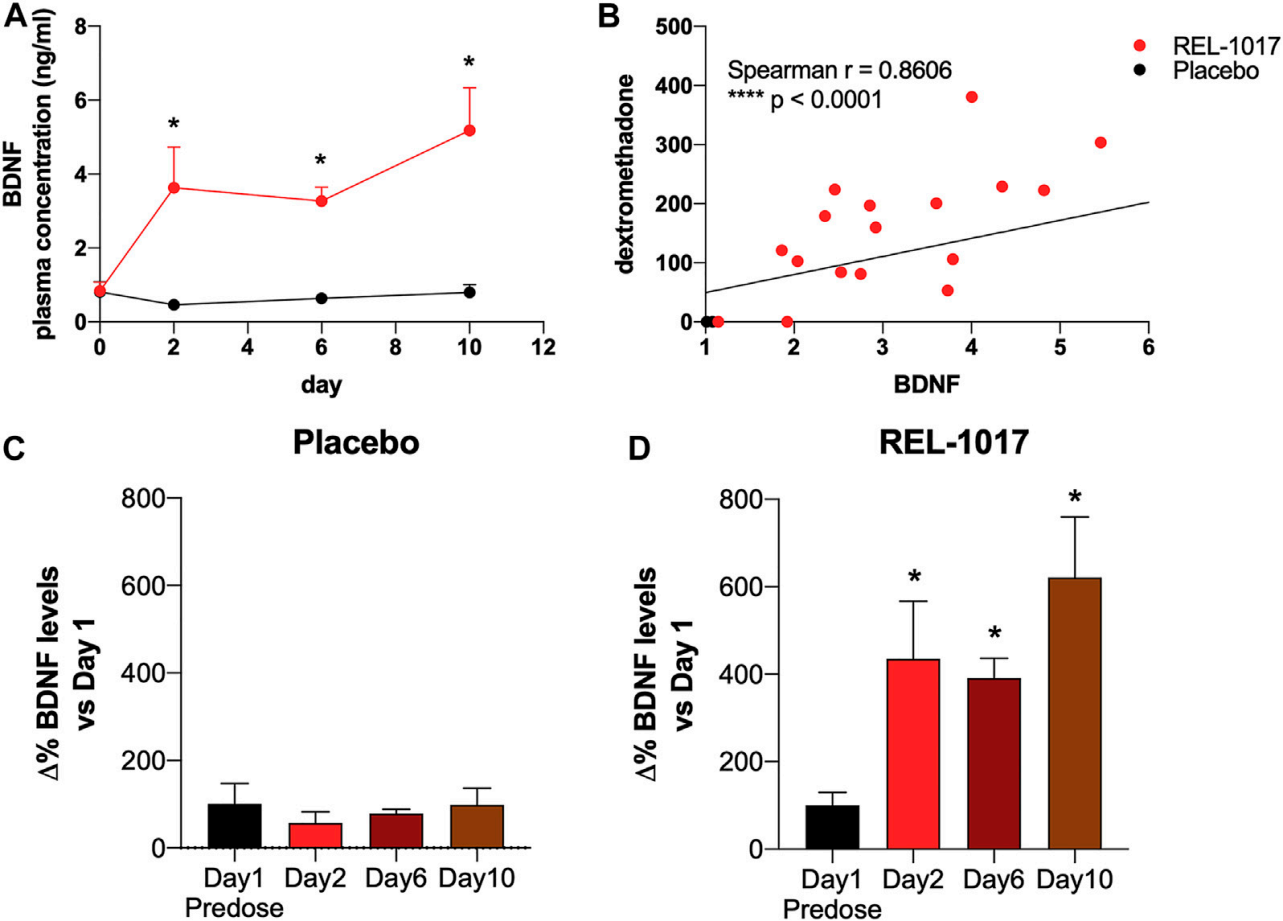
**(A)** Plasma BDNF concentration in placebo (black line) and REL-1017-treated (red line) subjects. *p* < 0.05 vs. placebo at the same time point. **(B)** Spearman correlation between REL-1017 and BDNF plasma concentrations. **(C)** Percentage changes in BDNF levels in placebo subjects. **(D)** delta changes in BDNF levels in treated subjects. *p < 0.05 vs. Day 1 (baseline).

**FIGURE 2:**
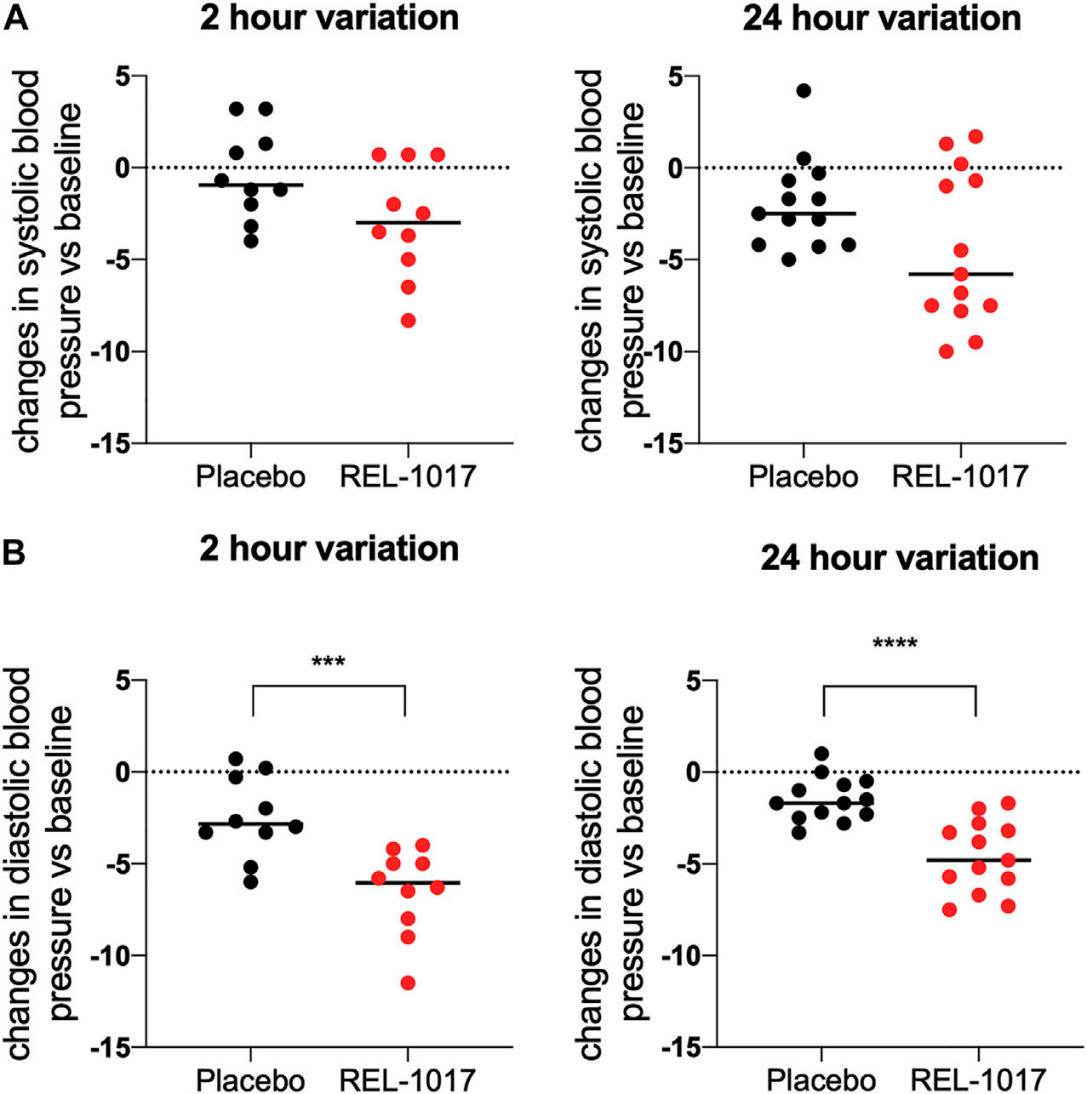
Changes in systolic **(A)** and diastolic **(B)** blood pressure 2 and 24 h after REL-1017 administration. The data represent differences from baseline in BP measurements taken during treatment. ***p < 0.001 and ****p < 0.0001 vs. placebo.

BDNF levels were significantly higher in REL-1017-treated subjects compared to the placebo group starting on Day 2 (*p* = 0.0357 vs. placebo); no significant difference between the two groups were present at baseline (Figure 1A). We also observed in all REL-1017-treated subjects an increase in BDNF plasma levels post-treatment compared to Day 1 pre-treatment values. The robust increase in BDNF plasma levels was maintained throughout the duration of treatment with REL-1017 (*p* = 0.028 at Day 2, *p* = 0.043 at Day 6, and *p* = 0.028 at Day 10 vs. pre-treatment, respectively). By contrast, BDNF plasma levels remained unchanged in the placebo group (Figures 1C,D). Plasma BDNF levels were significantly correlated with plasma levels of REL-1017 (*p* < 0.0001, Figure 1B). The lowest Day 10 plasma BDNF increase (less than twice the pre-treatment level) was seen in study subject with the lowest Day 10 REL-1017 level and AUC among all six treated subjects (Subject 6, Supplementary Table S1). We also observed significant changes in systolic and diastolic BP at 2 and 24 h after administration of REL-1017. While the decrease in systolic BP did not reach statistical significance (Figure 2A), a highly significant decrease in diastolic BP was evident in REL-1017 vs. placebo subjects (Figure 2B), both at 2 and 24 h after drug administration.

## Discussion

Healthy volunteers confined to an inpatient unit for a phase 1 clinical study are likely subjected to significant psychological stress. In these subjects, psychological stress could activate NMDAR in specific brain circuits, and NMDAR hyperactivation may in turn lead to dysregulated calcium signaling with downregulation of the synthesis and/or release of BDNF, as seen in rodent models of depressive-like behavior (Fogaça et al., 2019). Administration of REL-1017 in animal models rapidly reverses depressive like behavior (Fogaça et al., 2019; Hanania et al., 2020), *via* BDNF dependent mechanisms (Fogaça et al., 2019) and produced a rapid and robust increase in plasma BDNF in subjects confined to an inpatient for a phase 1 study by nearly 3-fold at Day 2 and Day 6 and more than 5-fold at Day 10. The role of BDNF in the cardiovascular system has been studied extensively and convincing evidence points to BDNF as a protective hormone in cardiovascular homeostasis (Pius-Sadowska and Machaliński, 2017). Furthermore, while the neuropsychiatric phenotypes of individuals carrying the Val66Met SNP have been studied extensively (Glatt and Lee, 2016), the consequences of this polymorphism on cardiovascular function are not completely defined. However, clinical studies demonstrated that subjects carrying Val/Val genotype have a higher risk than Met allele carriers of developing cardiovascular events (Jiang et al., 2017), further strengthening the link between BDNF and cardiovascular health.

Interestingly, BP was reduced in response to REL-1017 treatment. These effects may be due to the blocking effect of REL-1017 on NMDAR channels on neurons in the ventral medial prefrontal cortex, which physiologically activate the cardiac baroreflex response (Lagatta et al., 2018). Alternatively, the hypotensive effect of REL-1017 may be related to NMDAR blockade of neurons that are part of circuits linked to BDNF-dependent antidepressant effects. Val66Met polymorphism has been associated with higher anticipatory cortisol stress response and anxiety in healthy adults (Colzato et al., 2011).

It should be underscored that the data reported in this study were obtained from a retrospective analysis of a limited number of subjects. However, the BDNF increase in treated subjects was quite dramatic. Furthermore, these clinical findings are consistent with preclinical studies demonstrating that REL-1017 exerts an antidepressant-like activity in animal models of depressive-like behavior comparable to that exerted by ketamine and that this antidepressant-like effect may be due to the modulation of neural plasticity *via* BDNF-dependent mechanisms (Fogaça et al., 2019).

In conclusion, given the demonstrated preclinical efficacy of REL-1017 in murine models of depressive-like behavior and the overall very favorable pharmacokinetic, safety, and tolerability profile from two phase 1 studies (Bernstein et al., 2019), including the lack of psychotomimetic and opioidergic side effects, REL-1017 may represent a true breakthrough for the treatment of depression. The increase in BDNF plasma levels after REL-1017 administration in subjects experiencing a stressful event, could help clarify the molecular mechanisms underlying the therapeutic efficacy of REL-1017 and other NMDAR channel blockers and the pathophysiology of MDD.

## Data Availability

The original contributions presented in the study are included in the article/Supplementary Material, further inquiries can be directed to the corresponding author.
