# Supplementary material for: REL-1017 (Esmethadone) Increases Circulating BDNF Levels in Healthy Subjects of a Phase 1 Clinical Study
Source: Front Pharmacol. 2021 Apr 28;12:671859. doi: 10.3389/fphar.2021.671859 (PMC8113752; doi:10.3389/fphar.2021.671859)
Supplement: Supplementary file 1 [file Table1.pdf]

Supplementary Table 1: REL-1017 AUC_{0-10} and difference between pretreatment and day 10 BDNF levels.

Subject	REL-1017 AUC ng ·d/ml	Δ BDNF (day 10-day1)
REL-1017 Subject 1	2533	2.861
REL-1017 Subject 2	1625	10.085
REL-1017 Subject 3	1343	2.447
REL-1017 Subject 4	1537	3.887
REL-1017 Subject 5	1924	4.962
REL-1017 Subject 6	774	1.811
Placebo Subject 1	0	-0.074
Placebo Subject 2	0	0.035
